# Bilateral tympanokeratomas (cholesteatomas) with bilateral otitis media, unilateral otitis interna and acoustic neuritis in a dog

**DOI:** 10.1186/s13028-018-0386-4

**Published:** 2018-05-22

**Authors:** Liv Østevik, Kathrine Rudlang, Tuva Holt Jahr, Mette Valheim, Bradley Lyndon Njaa

**Affiliations:** 1 Department of Basic Sciences and Aquatic Medicine, Faculty of Veterinary Medicine and Biosciences, Norwegian University of Life Sciences, Ullevålsveien 72, 0454 Oslo, Norway; 2 Department of Companion Animal Clinical Sciences, Faculty of Veterinary Medicine and Biosciences, Norwegian University of Life Sciences, Ullevålsveien 72, 0454 Oslo, Norway; 3 Section of Pathology, Department of Laboratories, Norwegian Veterinary Institute, Ullevålsveien 68, 0454 Oslo, Norway; 4 Department of Diagnostic Medicine/Pathobiology, Kansas State Veterinary Diagnostic Laboratory, College of Veterinary Medicine, Kansas State University, K-221 Mosier Hall, Manhattan, KS 66506-5802 USA; 5 Present Address: Fish Vet Group Norge, Hoffsveien 21-23, 0275 Oslo, Norway

**Keywords:** Cholesteatoma, Dog, Ear, Meningitis, Otitis interna, Tympanokeratoma

## Abstract

**Background:**

An aural cholesteatoma, more appropriately named tympanokeratoma, is an epidermoid cyst of the middle ear described in several species, including dogs, humans and Mongolian gerbils. The cyst lining consists of stratified, keratinizing squamous epithelium with central accumulation of a keratin debris. This case report describes vestibular ganglioneuritis and perineuritis in a dog with chronic otitis, bilateral tympanokeratomas and presumed extension of otic infection to the central nervous system.

**Case presentation:**

An 11-year-old intact male Dalmatian dog with chronic bilateral otitis externa and sudden development of symptoms of vestibular disease was examined. Due to the dog’s old age the owner opted for euthanasia without any further examination or treatment and the dog was submitted for necropsy. Transection of the ears revealed grey soft material in the external ear canals and pearly white, dry material consistent with keratin in the tympanic bullae bilaterally. The brain and meninges were grossly unremarkable. Microscopical findings included bilateral otitis externa and media, unilateral otitis interna, ganglioneuritis and perineuritis of the spiral ganglion of the vestibulocochlear nerve and multifocal to coalescing, purulent meningitis. A keratinizing squamous epithelial layer continuous with the external acoustic meatus lined the middle ear compartments, consistent with bilateral tympanokeratomas. Focal bony erosion of the petrous portion of the temporal bone and squamous epithelium and Gram-positive bacterial cocci were evident in the left cochlea. The findings suggest that meningitis developed secondary to erosion of the temporal bone and ganglioneuritis and/or perineuritis of the vestibulocochlear nerve.

**Conclusions:**

Middle ear tympanokeratoma is an important and potentially life-threatening otic condition in the dog. Once a tympanokeratoma has developed expansion of the cyst can lead to erosion of bone and extension of otic infection to the inner ear, vestibulocochlear ganglion and nerve potentially leading to bacterial infection of the central nervous system.

**Electronic supplementary material:**

The online version of this article (10.1186/s13028-018-0386-4) contains supplementary material, which is available to authorized users.

## Background

An aural cholesteatoma, more appropriately named tympanokeratoma [1] is a keratin cyst of the middle ear reported in several species, including dogs [2], humans [3] and Mongolian gerbils [4]. The cyst lining consists of stratified, keratinizing squamous epithelium with central accumulation of a keratin debris [3]. The term “cholesteatoma” is a misnomer as the cyst does not contain cholesterol nor it is a neoplasm. Once a tympanokeratoma is formed, it can expand, destroy the surrounding bone tissue of the middle ear and lead to neurological abnormalities. In dogs, tympanokeratomas are most often concurrent with chronic otitis media and externa [5–9]. Canine tympanokeratomas are most frequently unilateral, but two reports of bilateral tympanokeratomas in dogs exist [8, 9]. Histology is necessary to confirm the diagnosis. In most reports only tissue resected during surgery is available, thus histological evaluation of the remainder of the ear has frequently not been performed [6, 7, 10, 11].

Abundant keratin debris in the middle ear may allow bacteria to form biofilms and persist in spite of antibiotic treatment [12]. In a minority of humans with chronic otitis media, infection can spread to involve the central nervous system (CNS), and extracranial and intracranial complications of middle ear disease are reported to be more common in patients with concurrent tympanokeratomas [13, 14]. Presumed extension of otic infection to the CNS has recently been reported in a dog with chronic otitis externa and tympanokeratoma, where meningitis was diagnosed after cytological examination of cerebrospinal fluid [11]. In this report, a Dalmatian dog with histologically verified chronic bilateral otitis externa and media, bilateral tympanokeratomas and unilateral otitis interna and acoustic ganglioneuritis presumably leading to meningitis is described. Extension of middle ear infection associated with tympanokeratomas to the inner ear and the CNS has been described in dogs previously [6, 11, 15, 16]. However, histopathological evidence of tympanokeratomas causing erosion of the temporal bone and leading to secondary bacterial vestibular ganglioneuritis or perineuritis has not been reported.

## Case presentation

An 11-year-old intact male Dalmatian dog was presented with a complaint of intermittent ataxia and lethargy after surgical removal of a skin tumour 10 days earlier. The dog’s clinical signs included a slightly elevated rectal temperature, moderate depression, marked bilateral iris atrophy and reduced indirect and direct pupillary light responses. Both pinnae were thickened and fibrotic with brown exudate in the external acoustic meatus, consistent with chronic otitis externa. Elleven hours later, the dog had difficulty maintaining balance, displayed spontaneous nystagmus, seemed to be circling to the left, was ataxic and had lost positional reflexes in both hind limbs. A tentative diagnosis of vestibular syndrome was made, but due to the dog’s old age, the owner opted for euthanasia without any further examination or treatment.

Additional historical information included chronic atopic dermatitis and intermittent otitis externa since the age of 6 months. Topical treatment had been administered intermittently and included antibiotics (Fucidin, Polymyxin B), antifungal drops (Miconazole) and unspecified corticosteroids. In addition, the dog has been treated with low dose oral prednisolone for the past 6 years.

**Fig. 1:**
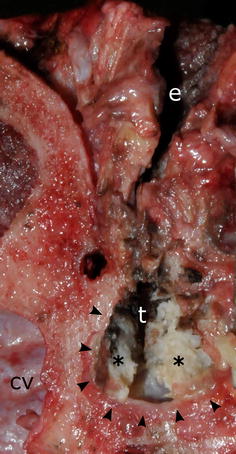
Keratin debris in the left middle ear. Transection of the left ear shows grey, soft material in the external ear canal (e) and pearly white keratin (asterisk) in the tympanic bulla (t). To the left of the image is the cranial vault (cv). The tympanic bulla is formed by the petrous portion of the temporal bone (arrowheads)

At necropsy, a grey soft material was present in the external ear canals and pearly white, dry material consistent with keratin was found in the tympanic bullae bilaterally (Fig. 1). Grey-white, firm, fibrous tissue partially obliterated the right bulla. The brain and meninges were grossly unremarkable. Both kidneys had pitted, irregular cortices and granular cut surface, and urinary stones were found in the pelvis of the right kidney and urinary bladder. Swabs for bacteriological culturing were collected from the urinary bladder and the surgical skin-incision, but unfortunately not brain or ears.

The brain and tissues from the kidney, myocardium, intestine, liver and lung were collected for histology. Both ears were collected en bloc by sawing transactionally through the tympanic bullae and cranium. Tissue samples were fixed in 10% buffered formalin, and bone tissue was thereafter decalcified in 10% EDTA. The brain was sectioned transversely to include tissue from three levels of the cerebrum, just cranial to the optic chiasm, at the cranial aspect of the pituitary gland and caudally through the midbrain to include hippocampus and cerebral aqueduct. The cerebellum was sectioned in the midsagittal plane and then transversely to include the pons, while a single transverse section of the medulla of the brain stem was made. All samples were routinely processed, embedded in paraffin, sectioned at 2–4 μm and stained with haematoxylin and eosin. Serial microtomic sections of both ears and the caudal most section of the cerebrum were Gram stained and Periodic Acid-Schiff-stained. Immunohistochemistry for rabies virus antigen was performed with monospecific polyclonal rabbit serum N161-5 (Friedrich-Loeffler-Institut, Federal Research Institute for Animal Health, Germany). As negative controls serum from a non-immunized rabbit and a brain sections from a rabies-negative fox were used. Brain tissue from a rabies–positive reindeer served as a positive control. The slides were counterstained with haematoxylin, dehydrated, and mounted in aqueous medium (Aquatex; Merck, Darmstadt, Germany).

The external ear canals were stenotic with marked orthokeratotic hyperkeratosis, epidermal and ceruminous gland hyperplasia and dermal fibrosis expanding the otic integument. Ceruminous glands were dilated and contained mixed inflammatory infiltrates. Multifocal infiltrates of plasma cells, neutrophils, lymphocytes and macrophages were present in the dermis, while there were pustules, yeast cells and coccoid bacteria in the stratum corneum.

**Fig. 2:**
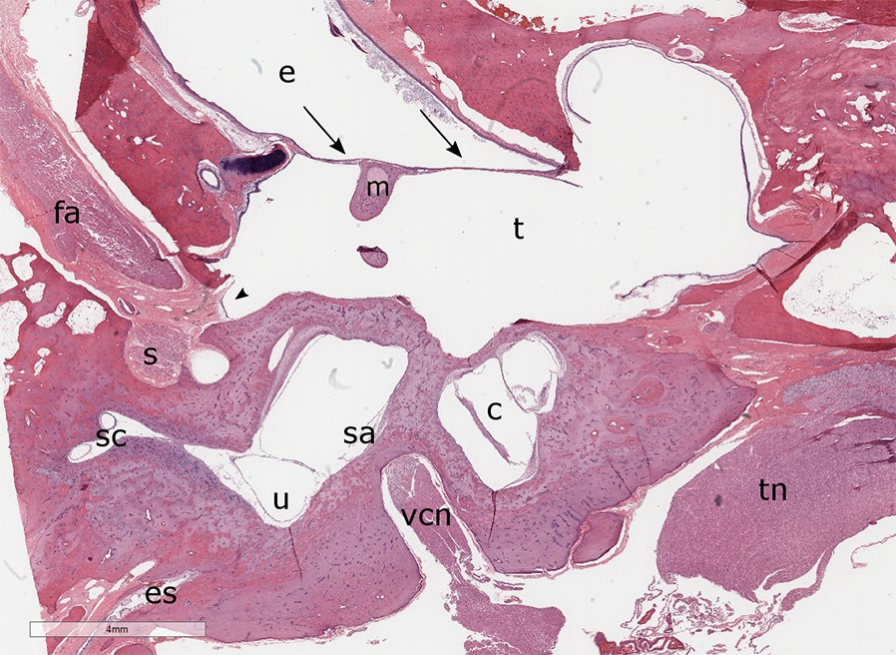
Subgross photomicrograph of a normal canine ear. An intact tympanic membrane (arrows) separates the external acoustic meatus (e) from the tympanic cavity (t). The tympanic cavity, a sagittal section of the cochlea (c), utriculus (u), and sacculus (sa) and vestibulocochlear nerve (vcn). The facial nerve (fa), the stapedius muscle (s), the facial canal foramen (arrowhead), semicircular canals (sc), endolymphatic sac (es) and manubrium (m) of the malleus are depicted in this image. The trigeminal nerve (tn) is present rostral to the cochlea. Note that the plane of section is different between Figs. 2 and 3. This section was collected from a 5-year-old German shepherd with heartworm. Haematoxylin and eosin stain, bar: 4 mm

**Fig. 3:**
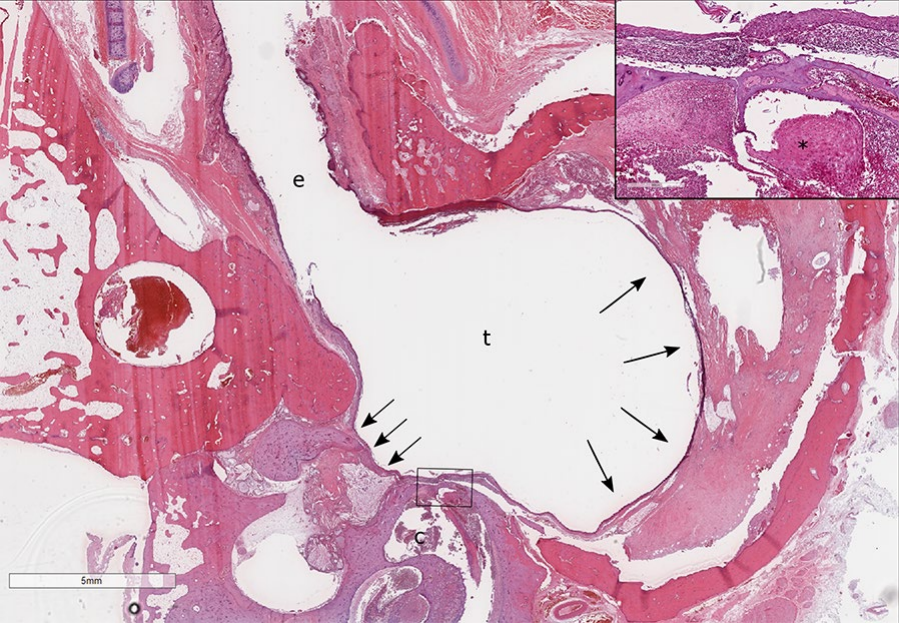
Subgross photomicrograph of the left ear. The tympanic membrane is not present resulting in direct communication between the external acoustic meatus (e) and tympanic cavity (t). A keratinizing squamous epithelial layer continuous with the external acoustic meatus (arrows) lines the middle ear compartment. Inset (square): A large aggregate of squamous epithelium (asterisk) is present within the cochlea (c) mixed with neutrophilic inflammation. This supports erosion of the petrous portion of the temporal bone and extension of the tympanokeratoma through this defect in the bone. Haematoxylin and eosin stain, bar: 5 mm

Compared to a normal canine ear (Fig. 2) marked histopathological changes were found in the middle ears of this dog. Both left and right tympanic membranes were unapparent, and there was direct communication between the external acoustic meatus and tympanic cavity. A keratinizing squamous epithelial layer continuous with the external acoustic meatus lined the middle ear compartment consistent with bilateral tympanokeratomas (Fig. 3). Abundant laminar keratin and bacterial cocci were seen in the lumen of the right tympanic bulla. Additionally, the right bulla was filled by mature, dense, fibrous tissue containing multiple glands lined by a single layer of ciliated respiratory epithelium mixed with goblet cells. Marked mucoperiosteal fibrosis, pseudogland formation and cholesterol clefts with mixtures of neutrophils and macrophages were found within the remainder of middle ear compartment.

**Fig. 4:**
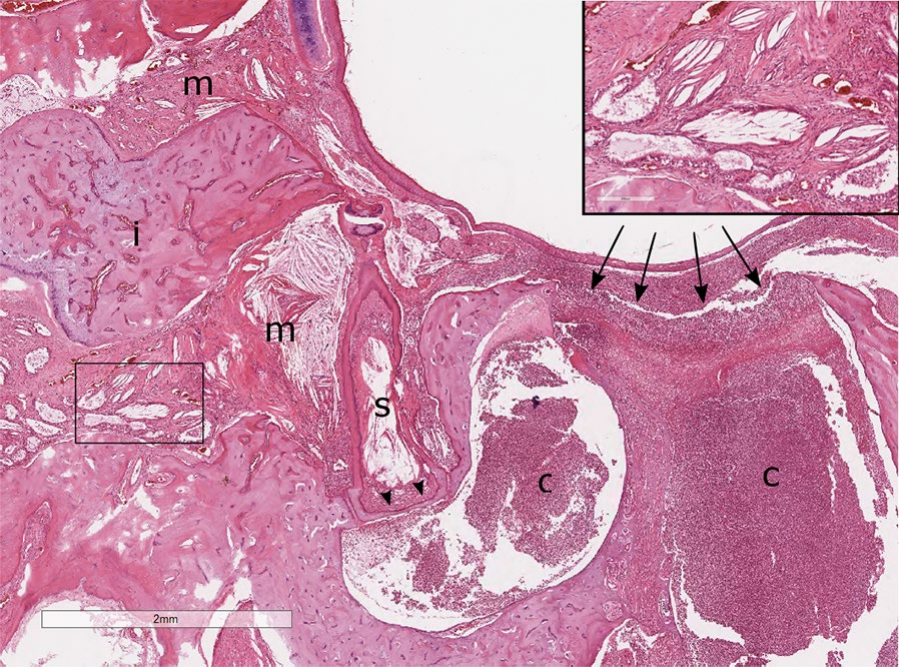
Photomicrograph of the tympanokeratoma eroding the temporal bone. In the left ear focal bony erosion of the petrous portion of the temporal bone is evident subjacent to the tympanokeratoma (arrows). Abundant inflammatory cells are present in the cochlea (c). Marked mucoperiosteal fibrosis, pseudogland formation and collections of cholesterol clefts are found within the remainder of middle ear compartment (m) surrounding the incus (i) and stapes (s). The stapes remains anchored in the vestibular (oval) window (arrowheads) supporting spread to the internal ear did not occur through the vestibular window. Inset (square): Marked mucoperiosteal fibrosis, pseudogland formation and collections of cholesterol clefts in the middle ear. Haematoxylin and eosin stain, bar: 2 mm

**Fig. 5:**
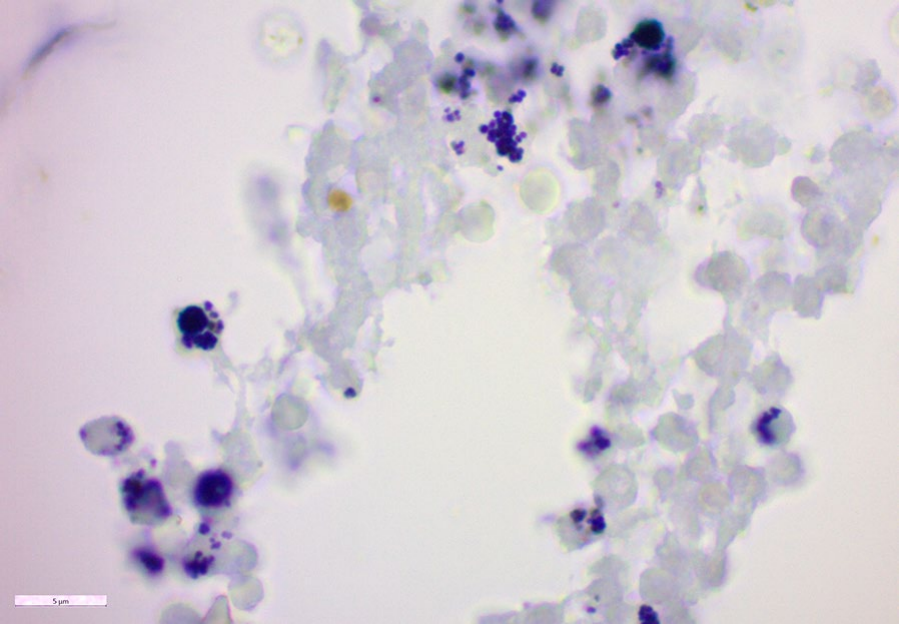
Photomicrograph demonstrating bacteria in the inner ear. Abundant intracellular and extracellular Gram-positive bacterial cocci forming clusters were found in the left cochlea. Gram stain, bar: 5 μm

**Fig. 6:**
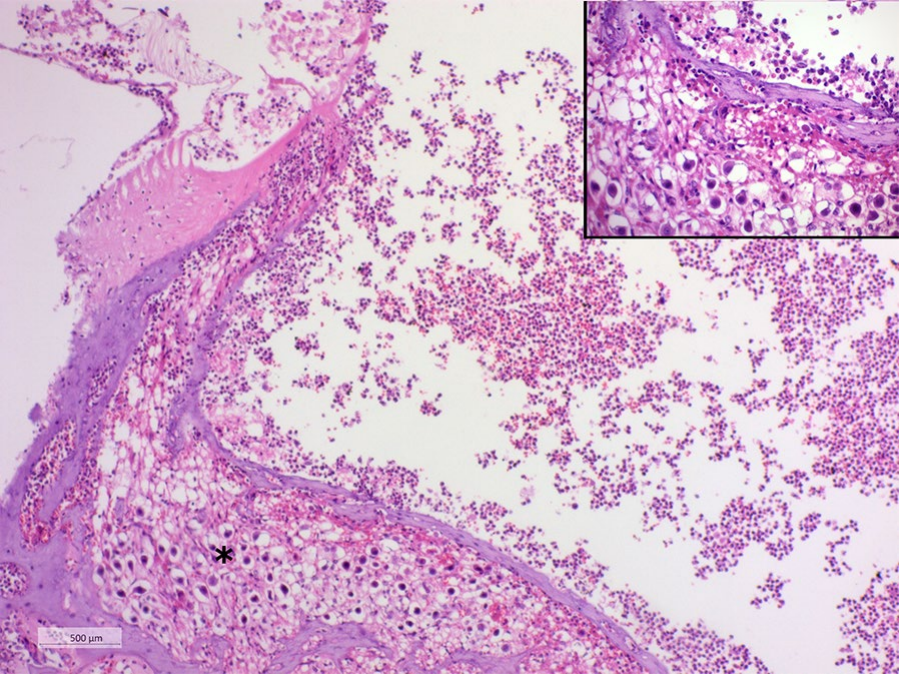
Photomicrograph demonstrating suppurative inflammation in the inner ear. Infiltrates of neutrophils, necrotic debris and haemorrhage are present in the left cochlea and spiral ganglion (asterisk). Inset: Infiltrates of neutrophils and haemorrhage in the spiral ganglion. Haematoxylin and eosin stain, bar: 500 μm

In the left ear focal, complete bony erosion of the petrous portion of the temporal bone was present adjacent to the tympanokeratoma (Fig. 4). An aggregate of keratinized squamous epithelial cells, marked infiltrates of neutrophils and multiple Gram-positive bacterial cocci were observed in the left cochlea (Fig. 5). The inflammation extended to the spiral ganglion of the vestibulocochlear nerve (cranial nerve VIII) and inflammatory infiltrates and haemorrhage were observed within the spiral ganglion and in the surrounding bone tissue (Fig. 6). There was no evidence of extension of infection through the vestibular (oval) window, but involvement of the cochlear (round) window could neither be confirmed nor excluded.

**Fig. 7:**
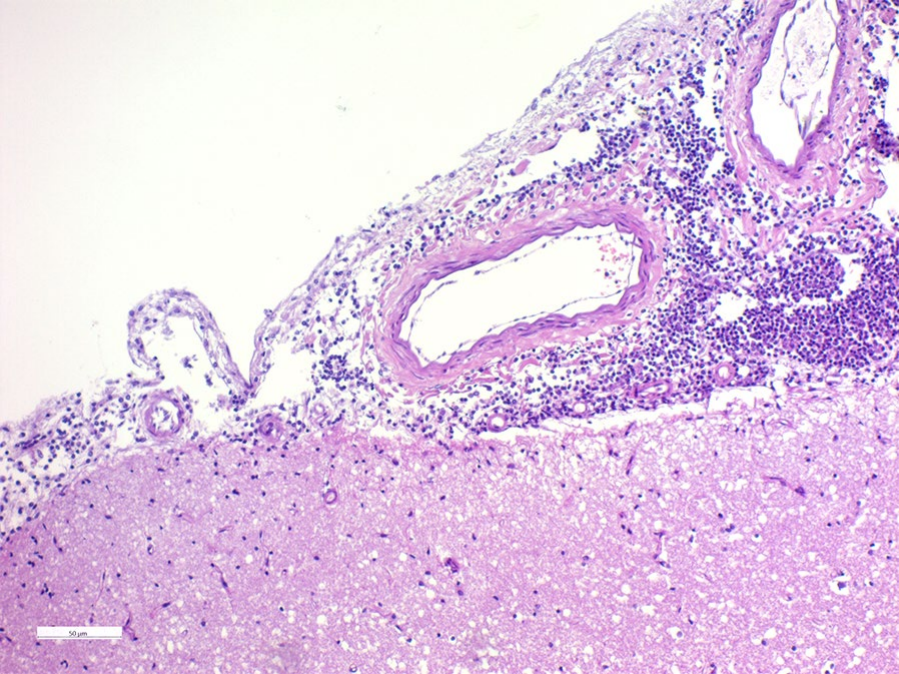
Photomicrograph demonstrating suppurative meningitis. In the meninges were multifocal to coalescing, inflammatory infiltrates of neutrophils, lymphocytes, macrophages and plasma cells, suggestive of bacterial meningitis. Haematoxylin and eosin stain, bar: 50 μm

Multifocal to coalescing inflammatory infiltrates of neutrophils, lymphocytes, macrophages and plasma cells suggestive of bacterial meningitis was found in the meninges of all examined sections from the brain, including, cerebrum, cerebellum and brain stem (Fig. 7). However, bacteria were not identified in the Gram-stained brain section. Eosinophilic, round to oval, intracytoplasmic inclusions were found in neurons of the thalamus and in Purkinje cells of the cerebellum (see Additional file 1 for photomicrograph). Inclusions were PAS-negative and negative for rabies virus antigen most consistent with rabies-like inclusions. A mixture of bacteria dominated by *Staphylococcus pseudintermedius* was cultured from the urinary bladder, while no bacteria grew from the surgical incision. Additional diagnoses included chronic, interstitial, lymphoplasmacytic nephritis and glomerulosclerosis, mild eosinophilic enteritis and mild, interstitial, lymphohistiocytic pneumonia.

## Discussion and conclusions

The finding of keratinizing squamous epithelium in both middle ears of this dog is characteristic of tympanokeratoma. The presence of inflammatory cells and bacteria in the middle and internal ear compartments and spiral ganglion of the vestibulocochlear nerve confirm the diagnoses of concurrent bilateral otitis externa and media, unilateral otitis interna and bacterial infection. While an extension of the bacterial infection from the ear to the meninges was not confirmed, it is likely that the vestibulocochlear nerve or perineuronal tissue served as a pathway for bacterial spread to the meninges.

The eosinophilic intracytoplasmic inclusions in the thalamic neurons and cerebellar Purkinje cells were rabies-antigen and PAS negative thus excluding a concurrent rabies virus infection. Canine rabies-like inclusions have been characterized by Nietfield et al. [17] and were consisting of lamellar bodies, stacks of closely packed membranous cisterns, and amorphous granular material. Rabies-like inclusions have been identified in dogs with and without clinical signs of neurologic disease and mild inflammatory infiltrates were found in only one of seven reported dogs [17]. Thus, these inclusions are of uncertain significance and may be an incidental finding in this case.

Canine tympanokeratomas most commonly develop in animals with a median age of 8 years [6]. In dogs as in humans, males are overrepresented [5, 6, 8, 18]. The prevalence of tympanokeratoma in dogs is largely unknown. A study examining histopathological findings in dogs with middle ear disease identified tympanokeratoma in 7 of 62 ears examined (11%) [2]. Presenting clinical signs in dogs with tympanokeratoma may include ataxia, head tilt, nystagmus, circling, facial paralysis and pain when opening the jaw [5, 6, 8]. In addition, clinical signs of otitis externa are present and most dogs have a long history of otitis externa [6, 8]. Clinical signs of vestibular disease could be caused by both bacterial infection of the middle and internal ear and osseous resorption and destruction following cyst expansion. In this case circling, ataxia and nystagmus are suggestive of unilateral, peripheral vestibular disease and are likely due to the spread of infection to the left cochlea and vestibulocochlear nerve [19]. However, ataxia and nystagmus can be observed in both central and peripheral vestibular syndrome and differentiating peripheral from central vestibular syndrome can be challenging. Depressed mentation, generalized proprioceptive ataxia and conscious proprioceptive deficits are suggestive of central vestibular disease [19]. Proprioceptive deficits were observed in the current case, but these were limited to the hind limbs and not generalized.

Extension of infection from the ear to the meninges and/or CNS seems to be uncommon in dogs with otitis media. Such extension has been reported in six dogs with otitis media [15, 16, 20, 21], three dogs with tympanokeratoma and otitis media [11, 15, 16], and two dogs with postsurgical recurrence of tympanokeratomas [6]. Cerebrospinal fluid cytology and bacteriology, computed tomography and magnetic resonance imaging have been used to diagnose involvement of the CNS and meninges, but detailed histologic examination of the ear or brain were not reported in any of the cases with cholesteatoma [6, 11, 15, 16, 20, 21]. Unfortunately, samples for bacterial culture from the ears or meninges were not collected from the current case. However, Gram-positive cocci in the cochlea and tympanic bulla confirm bacterial infection of the middle and internal ear and suggests an ascending infection to the meninges.

The pathogenesis of acquired tympanokeratoma remains controversial and researchers have proposed four major theories. Suggested origins include (1) tympanic membrane invagination into the middle ear, (2) external ear canal squamous epithelium that migrates into the middle ear, (3) metaplastic transformation of middle ear respiratory epithelium or (4) hyperplasia and cyst formation in the basal layer of the pars flaccida of the tympanic membrane [22]. However, Yamamoto-Fukada et al. [23] showed that the tympanic membrane is the origin of the cholesteatoma epithelium using a gerbil model of cholesteatoma. All theories suggest that chronic inflammatory stimuli and bacterial infection are important factors for development and continued growth of tympanokeratomas [18, 22]. Additionally, some researchers suggest a combination of processes is involved in the formation and growth of tympanokeratomas [22].

Tympanokeratomas in dogs are most often associated with chronic otitis media and presumably develop secondary to chronic otitis and tympanic membrane retraction. Once a tympanokeratoma has developed, infection and tympanokeratoma growth can potentially act synergistically. Keratin debris from the tympanokeratoma may allow bacteria to form biofilm and persist in spite of antimicrobial treatment [12] and inflammatory stimuli and bacterial infection could promote cyst growth and expansion [18, 22]. The long-term treatment with immunosuppressive corticosteroids may also have predisposed this particular dog for both persistence and potentially spread of the otic infection.

Four pathways for bacterial spread from the middle ear to the CNS exists and includes; (1) bone erosion and destruction, (2) direct extension through natural pathways (vestibular or cochlear window, cochlear aqueduct or endolymphatic duct and sac), (3) thrombophlebitis of venules in the cranial bones and (4) haematogenous spread [24]. In the patient described here, the histological evidence of erosion of the petrous portion of the temporal bone with squamous epithelium present in the basal turn of the cochlea confirmed spread via bone erosion and destruction. Involvement of the cochlear (round window) could not be definitely confirmed or excluded, and this may have been an additional pathway of bacterial spread. From the internal ear further extension along the natural pathway of the cochlear aqueduct, vestibulocochlear nerve or both is most likely.

In conclusion, middle ear tympanokeratoma is an important and potentially life-threatening otic condition in the dog. Once a tympanokeratoma has developed, expansion of the cyst can lead to erosion of bone and extension of otic infection to the inner ear, vestibulocochlear ganglion and the central nervous system leading to bacterial infection of the CNS.

## Additional file

**Additional file 1.** Photomicrograph demonstrating neuronal inclusions. Eosinophilic, round to oval intracytoplasmic inclusions were found in neurons of the thalamus. Haematoxylin and eosin stain, bar: 5 μm.
