# Supplementary material for: Bilateral tympanokeratomas (cholesteatomas) with bilateral otitis media, unilateral otitis interna and acoustic neuritis in a dog
Source: Acta Vet Scand. 2018 May 22;60:31. doi: 10.1186/s13028-018-0386-4 (PMC5964671; doi:10.1186/s13028-018-0386-4)

Additional file 1.

Photomicrograph demonstrating neuronal inclusions.

Eosinophilic, round to oval intracytoplasmatic inclusions were found in neurons of the thalamus.

Haematoxylin and eosin stain, bar: 5 µm. (PDF 222 kb)

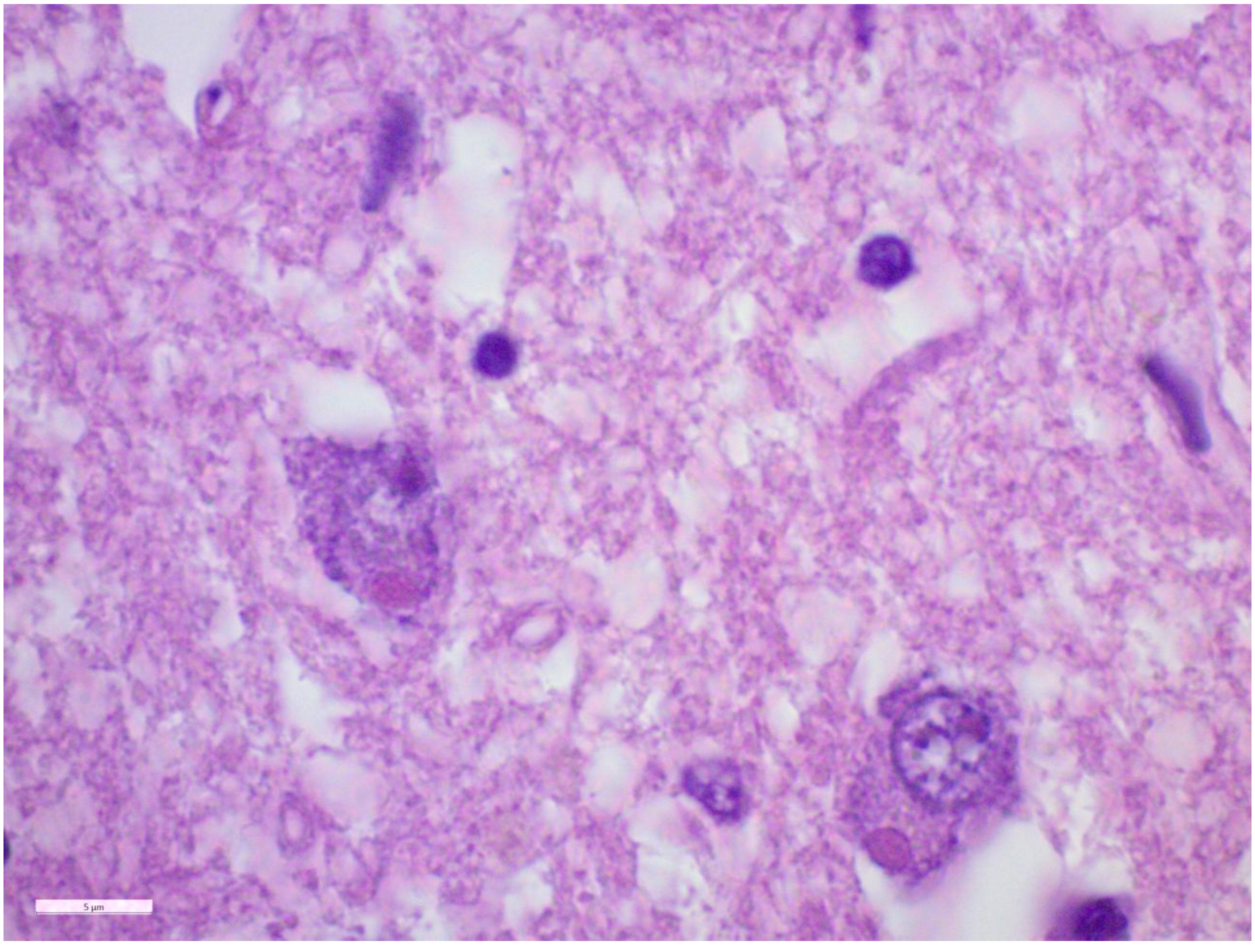

Supplement: Supplementary file 1 — Additional file 1. Photomicrograph demonstrating neuronal inclusions. Eosinophilic, round to oval intracytoplasmic inclusions were found in neurons of the thalamus. Haematoxylin and eosin stain, bar: 5 μm. [file 13028_2018_386_MOESM1_ESM.pdf]
